# Whole-exome sequencing of 81 individuals from 27 multiply affected bipolar disorder families

**DOI:** 10.1038/s41398-020-0732-y

**Published:** 2020-02-04

**Authors:** Andreas J. Forstner, Sascha B. Fischer, Lorena M. Schenk, Jana Strohmaier, Anna Maaser-Hecker, Céline S. Reinbold, Sugirthan Sivalingam, Julian Hecker, Fabian Streit, Franziska Degenhardt, Stephanie H. Witt, Johannes Schumacher, Holger Thiele, Peter Nürnberg, José Guzman-Parra, Guillermo Orozco Diaz, Georg Auburger, Margot Albus, Margitta Borrmann-Hassenbach, Maria José González, Susana Gil Flores, Francisco J. Cabaleiro Fabeiro, Francisco del Río Noriega, Fermin Perez Perez, Jesus Haro González, Fabio Rivas, Fermin Mayoral, Michael Bauer, Andrea Pfennig, Andreas Reif, Stefan Herms, Per Hoffmann, Mehdi Pirooznia, Fernando S. Goes, Marcella Rietschel, Markus M. Nöthen, Sven Cichon

**Affiliations:** 1 Centre for Human Genetics, University of Marburg, Marburg, Germany; 2 Institute of Human Genetics, University of Bonn, School of Medicine & University Hospital Bonn, Bonn, Germany; 3 Department of Biomedicine, University of Basel, Basel, Switzerland; 4 Department of Psychiatry (UPK), University of Basel, Basel, Switzerland; 5 Institute of Medical Genetics and Pathology, University Hospital Basel, Basel, Switzerland; 6 Department of Genetic Epidemiology in Psychiatry, Central Institute of Mental Health, Medical Faculty Mannheim, University of Heidelberg, Mannheim, Germany; 7 SRH University Heidelberg, Academy for Psychotherapy, Heidelberg, Germany; 8 Center for Lifespan Changes in Brain and Cognition (LCBC), Department of Psychology, University of Oslo, Oslo, Norway; 9 Department of Biostatistics, Harvard T.H. Chan School of Public Health, Boston, MA USA; 10 Cologne Center for Genomics, University of Cologne, Cologne, Germany; 11 Department of Mental Health, University Regional Hospital of Málaga, Institute of Biomedicine of Málaga (IBIMA), Málaga, Spain; 12 Unidad de Gestión Clínica del Dispositivo de Cuidados Críticos y Urgencias del Distrito Sanitario Málaga - Coin-Gudalhorce, Málaga, Spain; 13 Experimental Neurology, Department of Neurology, Goethe University Hospital, Frankfurt am Main, Germany; 14 Isar Amper Klinikum München Ost, kbo, Haar, Germany; 15 Department of Mental Health, University Hospital of Reina Sofia, Cordoba, Spain; 16 Department of Mental Health, Hospital of Jaén, Jaén, Spain; 17 Department of Mental Health, Hospital of Jerez de la Frontera, Jerez de la Frontera, Spain; 18 Department of Mental Health, Hospital of Puerto Real, Cádiz, Spain; 19 Department of Mental Health, Hospital Punta de Europa, Algeciras, Spain; 20 Department of Psychiatry, Carlos Haya Regional University Hospital, Malaga, Spain; 21 Department of Psychiatry and Psychotherapy, Medical Faculty, University Hospital Carl Gustav Carus, Technische Universität Dresden, Dresden, Germany; 22 Department of Psychiatry, Psychosomatic Medicine and Psychotherapy, University Hospital Frankfurt am Main, Frankfurt am Main, Germany; 23 Institute of Neuroscience and Medicine (INM-1), Research Center Jülich, Jülich, Germany; 24 Department of Psychiatry and Behavioral Sciences, Johns Hopkins University School of Medicine, Baltimore, MD USA

**Keywords:** Molecular neuroscience, Genetics

## Abstract

Bipolar disorder (BD) is a highly heritable neuropsychiatric disease characterized by recurrent episodes of depression and mania. Research suggests that the cumulative impact of common alleles explains 25–38% of phenotypic variance, and that rare variants may contribute to BD susceptibility. To identify rare, high-penetrance susceptibility variants for BD, whole-exome sequencing (WES) was performed in three affected individuals from each of 27 multiply affected families from Spain and Germany. WES identified 378 rare, non-synonymous, and potentially functional variants. These spanned 368 genes, and were carried by all three affected members in at least one family. Eight of the 368 genes harbored rare variants that were implicated in at least two independent families. In an extended segregation analysis involving additional family members, five of these eight genes harbored variants showing full or nearly full cosegregation with BD. These included the brain-expressed genes *RGS12* and *NCKAP5*, which were considered the most promising BD candidates on the basis of independent evidence. Gene enrichment analysis for all 368 genes revealed significant enrichment for four pathways, including genes reported in de novo studies of autism (*p*_adj_ < 0.006) and schizophrenia (*p*_adj_ = 0.015). These results suggest a possible genetic overlap with BD for autism and schizophrenia at the rare-sequence-variant level. The present study implicates novel candidate genes for BD development, and may contribute to an improved understanding of the biological basis of this common and often devastating disease.

## Introduction

Bipolar disorder (BD) is a complex neuropsychiatric disorder characterized by recurrent episodes of mania and depression. BD has a lifetime prevalence of 1%, and an estimated heritability of ~60–85%^1–3^. The World Health Organization ranks BD among the largest contributors to the global burden of disease^3^.

Genetic linkage studies, candidate gene studies, and genome-wide association studies (GWAS) have generated initial insights into the genetic architecture of BD. Recent GWAS have identified several common susceptibility loci for BD^4–9^. However, BD-driving pathways and networks remain largely unknown^10^. Models of BD are consistent with a polygenic contribution of common and rare variants to disease susceptibility^11^. Research has demonstrated that the cumulative impact of common alleles explains an estimated 25–38% of BD phenotypic variance^12,13^. Another substantial contribution to BD susceptibility is expected to come from rare variants^14^. A promising approach to the identification of rare, high-penetrance variants in BD is the investigation of large, multiply affected pedigrees. In these families, the existence of a co-segregating genetic variant of strong effect may be more likely than in sporadic patients^10,15^.

Initial whole-exome and whole-genome sequencing studies of BD patients have implicated a number of candidate genes. Preliminary results suggest an enrichment of rare genetic variants in: (i) specific gene sets, i.e., calcium signaling, axon guidance, cyclic adenosine monophosphate response element binding protein (CREB) signaling, potassium channels, and G protein-coupled receptors^16–21^; and (ii) genes that have been reported to harbor de novo nonsense and missense variants in studies of schizophrenia and autism^22^. Furthermore, a trio-based exome-sequencing study suggested that de novo variants may also be implicated in BD etiology, particularly, in patients with an early age of onset^23^. However, limited overlap in implicated genes is evident between studies, suggesting that the analysis of further pedigrees and samples is warranted before definitive conclusions can be drawn^10^.

The aim of the present study was to identify rare, high-penetrance susceptibility variants for BD via whole-exome sequencing (WES) in large BD pedigrees from Spain and Germany.

## Materials and methods

### Sample description

A total of 27 multigenerational, multiply affected Spanish (*n* = 23) and German (*n* = 4) families were investigated. A detailed description of the phenotypic assessment of the Spanish families is provided elsewhere^24^. In brief, the diagnostic assessment of affected and unaffected individuals in the Spanish families was performed using: the Schedule for Affective Disorders and Schizophrenia (SADS)^25^; the Operational Criteria Checklist for Psychotic Illness (OPCRIT)^26^; a review of medical records; and interviews with first and/or second-degree family members using the Family Informant Schedule and Criteria (FISC)^27^. Consensus best estimate diagnoses were assigned by two or more independent senior psychiatrists and/or psychologists, in accordance with the Diagnostic and Statistical Manual of Mental Disorders IV (DSM IV). In affected and unaffected members of the German families, phenotypic assessment and the assignment of diagnoses were performed by an experienced psychiatrist^28^.

No relationships were reported between the 27 individual families. In each of the 27 families, three individuals with BD were selected for WES (Supplementary Figure 1). These individuals were selected on the basis of being as distantly genetically-related as possible. The 81 selected individuals (37.0% male) had a DSM IV diagnosis of BD type I (*n* = 69); BD type II (*n* = 9); or BD not otherwise specified (NOS, *n* = 3).

The study was approved by the respective local ethics committees. Written informed consent was obtained from all participants prior to inclusion.

### WES

Library enrichment for WES was conducted using SureSelect^XT^Human All Exon v5 from Agilent Technologies (Santa Clara, CA, USA). Enriched samples were sequenced using an Illumina HiSeq2500 system (San Diego, CA, USA), and a 2 × 125 base pair (bp) paired end sequencing approach. Mean coverage of the sequences was 68.28×, and 96.90% of the sequencing reads had a coverage of >10×. Sequencing data were annotated according to the GRCh37/hg19 reference genome. The sequencing data are available upon request.

A detailed plan of the analytical steps is presented in the Supplement (Supplementary Figure 2). In a first step, separate analyses were conducted for each of the 27 families. For each of the three selected family members, Variant Calling Files were generated using the VARBANK pipeline (https://varbank.ccg.uni-koeln.de). The VARBANK pipeline integrates a number of publically available sequencing analysis tools. Among others, various GATK tools are used for diverse processing steps. The VARBANK pipeline is therefore based on GATK core components. VARBANK filter criteria were set for the detection of heterozygous variants (allele read frequency between 25% and 75%). Sequencing reads with a coverage of ≥10× were included in the subsequent analyses. The analyses focused on single-nucleotide variants/polymorphisms (SNVs, SNPs) and insertions or deletions (InDels) that: (i) resulted in an alteration in primary protein structure; or (ii) had strong splice site effects^29^. Only variants shared by all three investigated family members were included in the subsequent variant analysis, as these variants might be responsible for the exceptional aggregation of BD in the respective multiply affected families. The present rationale is based on the assumption that in multiply affected families, individual rare variants with a relatively strong effect (penetrance) on disease development may segregate. By concentrating solely on variants that were present in all three sequenced patients, the analysis focused on variants with a potentially high penetrance, and knowingly overlooked rare, disease-associated variants with lower penetrance. Although the term “segregation” is used in describing the exome-sequencing results, it should be noted that only “allele sharing” is actually observed. However, owing to the rarity of the identified variants, the observation of allele sharing between three exome-sequenced individuals from a given family is likely to reflect true segregation (i.e., identity-by-state = identity-by-descent).

The identified variants were filtered for a minor allele frequency (MAF) < 0.1% using the data of the Exome Aggregation Consortium (ExAC, http://exac.broadinstitute.org, release 0.3, non-psychiatric subsets)^30^. The majority of ExAC data originate from Europe (around 60%), and were thus considered appropriate in terms of estimating the MAF of variants identified in the present cohort.

To obtain functional predictions for the identified variants, the dbNSFP database was accessed. In accordance with Purcell et al.^31–33^, the five prediction tools SIFT, PolyPhen-2 HumDiv, PolyPhen-2 HumVar, LRT, and MutationTaster were used for the analysis of SNVs and SNPs. Only variants predicted to be potentially/probably damaging by at least three of the five prediction tools were included in the final list (Supplementary Table 1). For the analysis of InDels, the prediction tools MutationTaster and PROVEAN/SIFT were used. Only InDels predicted to be damaging by at least one of the three prediction tools were included in the subsequent analyses. Owing to their potential impact on protein function, nonsense variants that were classified as (probably) disease causing by the MutationTaster tool were included in the final list (Supplementary Table 1). For each of the identified variants, visual inspection of the sequencing reads in the VARBANK database was performed in order to control for technical artifacts.

### Kinship analysis

A kinship analysis was conducted for the 81 exome-sequenced individuals from the 27 multiplex families using the Sample Kinship analysis tool within the VARBANK pipeline. This tool determines the proportion of alleles that are shared between pairs of individuals. The thresholds for shared variant analysis are: MAF < 0.1%; target distance < 100 bp; and passing of GATK’s variant quality score recalibration filter. The resulting values are the number and percentage of shared alleles. The results report the pairwise comparison of one individual with all other individuals within the investigated cohort.

### Technical validation and extended segregation analyses

Using Sanger sequencing, technical validation and extended segregation analyses were performed for variants that were both: (i) rare, non-synonymous, and predicted to be potentially/probably damaging according to the above-mentioned criteria; and (ii) located in genes that harbored variants in at least two independent families. Extended segregation analysis was conducted in all family members (affected and unaffected) for whom DNA was available (Supplementary Figure 1). Primers for these experiments were designed using Primer3^34^. Cycle Sequencing was conducted with the BigDye Terminator v3.1. Sanger sequencing was conducted using an ABI3130 Genetic Analyzer (Life Technologies, Carlsbad, CA, USA). Primer sequences and PCR conditions are obtainable upon request.

### Rare variant association testing using RareIBD

RareIBD analyses were performed for the 16 rare variants that were investigated in the extended segregation analysis. RareIBD is a rare variant association method for large and extended pedigrees. RareIBD was selected as it is applicable to pedigrees with different family structures and those in which individuals in the top generations are missing^35^. RareIBD analysis (v1.2, http://genetics.bwh.harvard.edu/rareibd/) was conducted using the segregation analysis data of all family members for whom DNA was available. For the RareIBD analysis, individuals were defined as being affected if they were diagnosed with BD type I, BD type II, or BD NOS. All other individuals were defined as unaffected. RareIBD software settings were applied in accordance with the standard recommendations for the analysis. The resulting *p* values were Bonferroni-corrected for multiple testing according to the number of investigated variants (*n* = 16). For the quality control of the generated pedigree files, pedigrees were drawn using the CRAN-package kinship2, and the generated figures were then inspected to confirm correct family structure^36^.

### Brain expression of candidate genes

To determine whether candidate genes identified in the WES analyses are expressed in the human brain, the Genotype-Tissue Expression (GTEx) database was accessed (https://gtexportal.org/)^37^. The GTEx Portal comprises expression data from multiple brain regions. Using the GTEx data, average expression values were generated for 12 different brain regions (excluding the spinal cord). Genes with a mean expression of > 0.5 Reads Per Kilobase Million were considered brain-expressed.

### Investigation of identified candidate genes in published datasets

A literature search was conducted to determine whether candidate genes identified in the present study had been reported in previous independent next-generation sequencing studies of BD patients or in BD GWAS^8,9,18–20,22,23,38–42^.

### Gene set enrichment analysis

For the 368 genes that harbored rare, non-synonymous, and segregating variants, a systematic investigation of gene set enrichment was performed using the permutation-based method described in Goes et al.^22^, in order to account for potential confounders, such as coding length, sequencing coverage, and overall mutability. Testing was performed for an enrichment of genes reported in previous de novo studies of autism and schizophrenia, and genes encoding postsynaptic density (PSD) proteins or targets of the fragile X mental retardation protein (FMRP)^22^. In brief, curated gene lists were retrieved from studies that had summarized genes with de novo nonsense and missense variants in autism (*n* = 1781), and schizophrenia (*n* = 670), as well as genes encoding proteins found in the PSD (*n* = 1398) and the FMRP pathway (*n* = 795)^43,44^. Newly curated lists of de novo autism and schizophrenia gene sets were also included in the permutation-based gene set enrichment analysis. The novel autism gene set was downloaded from the database de novo-db (version 1.5) and annotated using the Variant Effect Predictor (VEP) tool^45,46^. Non-synonymous variants that showed an association with a primary autism phenotype were selected, resulting in a set of 3679 genes. The novel schizophrenia gene set was compiled from seven published whole-exome studies of schizophrenia trios^44,47–52^. All available exonic variants were combined and re-annotated using the VEP tool, which is based on the Gencode v19 genome build^46^. The novel schizophrenia data set comprised 714 genes with at least one de novo non-synonymous variant.

The permutation-based gene set enrichment analysis was also performed for 55 additional gene sets that had shown association with BD in previous sequencing studies or GWAS^9,16,17,20,21,53^. Where possible, the original pathway definitions given in the respective studies were used. When this was not possible, current pathway definitions were obtained from the databases Gene Ontology (GO, http://geneontology.org/); Kyoto Encyclopedia of Genes and Genomes (KEGG, https://www.genome.jp/kegg/); and Molecular Signatures Database (MSigDB, software.broadinstitute.org/gsea/msigdb)^54–59^.

Tests were then conducted to determine whether the 368 genes that harbored rare non-synonymous segregating variants in the present cohort were enriched for any of these 61 gene sets. The gene set enrichment analysis was also performed for 139 genes that harbored rare non-synonymous segregating variants, which were predicted to be potentially/probably damaging by all applied prediction tools.

An equal number of genes captured by the present WES study were selected at random and matched with our candidate genes for the following three potentially confounding metrics: cumulative exon length (± 20%); sequence coverage (± 20%); and a gene-specific measure of intolerance to missense variation (ExAC missense constraint *z* score). A total of 10,000 permutations were performed, and the number of times that randomly selected genes were found in each of the gene sets was counted. To obtain empirical *p* values, a comparison was made between the observed degree of overlap and gene sets with this null distribution.

The Benjamini & Hochberg method was applied to the resulting 122 *p* values in order to perform a false discovery rate correction. Adjusted *p* values of < 0.05 were considered statistically significant.

## Results

The kinship analysis revealed that individuals from different families shared a maximum of 3% of rare variants, which corresponds to a relationship that is more distant than second-degree cousin status. The analysis therefore confirmed the absence of close genetic relationships between the 27 investigated families.

WES revealed that a total of 378 rare, non-synonymous, and potentially functional variants were carried by all three investigated members in at least one family. These 378 variants spanned a total of 368 genes (Supplementary Table 1). Eight of these genes harbored rare segregating variants in at least two independent families (*ADGB, DCAF5, NCKAP5, PKHD1L1, AOAH, CAND2, DIDO1,* and *RGS12*; Table 1). All of the 16 rare variants detected in these eight genes were validated by Sanger sequencing (validation rate of 100%). Re-analysis of data from the GTEx database revealed that six of the eight genes are expressed in the human brain (Table 2). The two exceptions were *PKHD1L1* and *ADGB*.

**Table 1:** Summary of genes harboring rare segregating variants in at least two independent bipolar disorder (BD) families.

Gene	Family	Chr	Position	Ref	Alt	Mut Prot	dbSNP	MAF	SIFT	P2_HDIV	P2_HVAR	LRT	MutationTaster
*ADGB*	0014	6	147122366	C	T	p.R1529*		NA	NA	NA	NA	NA	D
	0085	6	147042628	G	T	p.W694C		NA	D	D	D	N	D
*AOAH*	0010	7	36661364	C	T	p.R202Q	rs373491776	2.88E-04	NA	D	D	D	D
	0012	7	36588239	A	G	p.I339T	rs144383001	3.64E-04	D	D	D	N	D
*CAND2*	0045	3	12868994	C	T	p.A1089V	rs200074296	8.89E-05	D	D	D	D	D
	0092	3	12856687	C	T	p.R352C		3.38E-05	D	D	D	D	D
*DCAF5*	0009	14	69520635	C	T	p.R923Q	rs751152443	1.11E-05	D	D	D	D	D
	1044	14	69520635	C	T	p.R923Q	rs751152443	1.11E-05	D	D	D	D	D
*DIDO1*	0022	20	61511484	G	A	p.P1942S		5.79E-05	D	D	P	U	D
	0041	20	61525253	C	T	p.G956R	rs143952489	9.20E-04	D	D	P	N	N
*NCKAP5*	0074	2	134275041	G	C	p.D19E		NA	D	D	D	NA	N
	0085	2	133539953	C	T	p.M1477I	rs182514291	9.99E-04	D	D	D	U	D
*PKHD1L1*	0014	8	110534504	A	T	p.N4041Y		NA	D	D	P	D	D
	0109	8	110527435	C	T	p.R3864C		3.70E-04	D	D	D	D	D
*RGS12*	0045	4	3319538	G	T	p.Q547H		NA	D	D	P	D	N
	0085	4	3318073	G	A	p.R59Q		2.21E-05	D	D	P	N	N

*Chr* chromosome, *Position* chromosomal position according to hg19/GRCh37, *Ref* reference allele, *Alt* alternative allele, *Mut Prot* alteration on the protein level, *MAF* minor allele frequency, *NA* not available, *P2* PolyPhen-2, *HDIV* HumDiv-trained PolyPhen-2 model^82^, *HVAR* HumVar-trained PolyPhen-2 model^82^, *LRT* likelihood ratio test, *D* damaging/probably damaging/deleterious/disease-causing, *P* possibly damaging, *N* neutral, *U* unknown.

**Table 2:** Overview of extended segregation analysis results.

Gene	Family	Chr	Position	Mut Prot	BD	Other psy dis	Unaffected	RareIBD	Brain exp
*ADGB*	0014	6	147122366	p.R1529*	3/3	1/2	0/1	>0.99	No
	0085	6	147042628	p.W694C	3/3	0/0	0/1	>0.99	
*AOAH*	0010	7	36661364	p.R202Q	4/4	0/0	4/6	>0.99	Yes
	0012	7	36588239	p.I339T	3/3	0/0	0/1	>0.99	
*CAND2*	0045	3	12868994	p.A1089V	4/4	1/1	3/4	>0.99	Yes
	0092	3	12856687	p.R352C	3/3	0/0	0/3	>0.99	
*DCAF5*	0009	14	69520635	p.R923Q	5/5	0/0	0/1	0.16	Yes
	1044	14	69520635	p.R923Q	5/6	1/1	1/3	>0.99	
*DIDO1*	0022	20	61511484	p.P1942S	4/4	0/2	4/5	>0.99	Yes
	0041	20	61525253	p.G956R	4/5	1/2	1/4	>0.99	
*NCKAP5*	0074	2	134275041	p.D19E	3/3	0/0	1/3	>0.99	Yes
	0085	2	133539953	p.M1477I	3/3	0/0	0/1	>0.99	
*PKHD1L1*	0014	8	110534504	p.N4041Y	3/3	0/2	0/1	0.49	No
	0109	8	110527435	p.R3864C	3/3	0/0	0/2	>0.99	
*RGS12*	0045	4	3319538	p.Q547H	4/4	1/1	1/4	>0.99	Yes
	0085	4	3318073	p.R59Q	3/3	0/0	0/1	>0.99	

Segregation analysis was conducted in all family members for whom DNA was available. Family members were divided into three categories: individuals affected with bipolar disorder (BD); individuals with other psychiatric disorders (Other psy dis); unaffected individuals (Unaffected). The number following the slash indicates the number of individuals with available DNA, the number before the slash indicates the number of individuals harboring the respective variant. Data on brain expression were accessed from the GTEx database.

*Chr* chromosome, *Position* chromosomal position according to hg19/GRCh37, *Mut Prot*, alteration on the protein level, *RareIBD* Bonferroni-corrected *p* value of the RareIBD analysis, *Brain exp* brain expression.

Extended segregation analysis of these 16 rare variants revealed that the variants in *ADGB*, *DCAF5*, *NCKAP5*, *PKHD1L1*, and *RGS12* might display high penetrance. In contrast, rare variants in *AOAH*, *CAND2*, and *DIDO1* were also detected in several unaffected family members, suggesting that these variants are less likely to be highly penetrant (Table 2).

In the RareIBD association analysis, two variants showed a nominally significant association with disease status: (i) variant p.N4041Y in the *PKHD1L1* gene (*p*_nom_ = 0.0305); and (ii) variant p.R923Q in *DCAF5* (family 0009, *p*_nom_ = 0.0097). However, neither of these associations withstood stringent Bonferroni correction for multiple testing (Table 2).

A total of 139 genes harbored rare, non-synonymous variants that were carried by all three affected family members and predicted to be potentially/probably damaging by all applied prediction tools. A broader list of rare segregating variants, which were predicted to be potentially/probably damaging by at least one of the applied tools, is provided in the Supplement (Supplementary Table 2).

Analyses were performed to determine whether any of the 368 genes with rare and potentially functional variants have been implicated in previous next-generation sequencing studies or GWAS of BD. This revealed a total of 19 overlapping genes. These included *ANK3*, which has been implicated in both BD GWAS and sequencing studies (Table 3).

**Table 3:** Overview of the analysis to determine whether any of the 368 genes with rare and potentially functional variants have been implicated in previous next-generation sequencing studies (NGS) or genome-wide association studies (GWAS) of bipolar disorder (BD).

Gene	Family	Chr	Position	Ref	Alt	Mut Prot	MAF	Category	Publications
*ANK3*	0215	10	61815451	G	A	p.H1828Y	2.22E-05	BD NGS, BD GWAS	Chen et al., 2013; Fiorentino et al., 2014; Georgi et al., 2014; Mühleisen et al., 2014; Stahl et al., 2019
*ANKRD39*	0085	2	97520089	C	G	p.G64R	1.11E-05	BD GWAS	Stahl et al., 2019
*BZRAP1*	0062	17	56389544	G	A	p.R880W	3.51E-05	BD NGS	Kataoka et al., 2016
*CABIN1*	0109	22	24468401	A	G	p.E858G	1.10E-04	BD NGS	Goes et al., 2016
*CUX1*	0023	7	101877499	A	G	p.M1201V	NA	BD NGS	Goes et al., 2016
*DPP3*	0041	11	66249825	A	G	p.T52A	NA	BD GWAS	Stahl et al., 2019
*MACF1*	0014	1	39893177	C	T	p.T3394I	4.30E-04	BD NGS	Kataoka et al., 2016
*MAU2*	0085	19	19454736	C	T	p.T355M	2.24E-05	BD GWAS	Stahl et al., 2019
*MYH7B*	0215	20	33583320	T	C	p.I1003T	4.47E-05	BD GWAS	Green et al., 2013b
*MYO10*	0074	5	16670681	A	G	p.L1946S	5.62E-05	BD NGS	Kataoka et al., 2016
*NISCH*	0023	3	52526224	G	A	p.R1414Q	4.50E-05	BD GWAS	Stahl et al., 2019
*OBSCN*	0012	1	228481095	G	A	p.A3637T	1.11E-05	BD NGS	Goes et al., 2016
*POC1A*	1044	3	52183400	C	A	p.G94V	NA	BD GWAS	Stahl et al., 2019
*PRKCZ*	1044	1	2075740	G	A	p.R171H	4.41E-04	BD NGS	Goes et al., 2016
*PROC*	0074	2	128186037	G	T	p.A301S	NA	BD NGS	Kataoka et al., 2016
*RIMS1*	0215	6	73108704	AAGAAGAAG	AAGAAG	p.K717del	NA	BD GWAS	Stahl et al., 2019
*SYNE1*	0014	6	152683306	T	G	p.K3433T	NA	BD GWAS	Green et al., 2013a
*TTBK2*	0085	15	43044464	C	A	p.D994Y	5.55E-05	BD GWAS	Stahl et al., 2019
*ZBTB24*	0215	6	109802434	C	T	p.D266N	3.31E-05	BD NGS	Goes et al., 2016

*Chr* chromosome, *Position* chromosomal position according to hg19/GRCh37, *Ref* reference allele, *Alt* alternative allele, *Mut Prot* alteration on the protein level, *MAF* minor allele frequency, *NA* not available.

Permutation-based gene set enrichment analyses for the 368 genes revealed significant enrichment for a total of four gene sets after correction for multiple testing (Table 4). Of the four gene sets investigated in Goes et al.^22^, a significant enrichment was found for the de novo autism gene set (81 observed vs. 44.4 expected, *p*_adj_ < 0.006, Table 4). Analyses of the larger, newly curated de novo autism and schizophrenia gene sets both revealed significant enrichment (*p*_adj_ < 0.006 and *p*_adj_ = 0.015, respectively; Table 4). Of the 55 additional gene sets that had shown association with BD in previous studies, only “Regulation of anatomical structure size” showed a significant enrichment in the present study (*p*_adj_ = 0.015).

**Table 4:** Overview of gene sets with a significant enrichment after correction for multiple testing in the enrichment analysis for the 368 genes with rare and potentially functional variants.

Significant gene sets	Size	*N* observed	*N* expected	*P* value	*P* value adj
de novo autism	1781	81	44.4	<0.0001	<0.006
de novo autism novel	3679	148	95.8	<0.0001	<0.006
de novo schizophrenia novel	714	34	18.3	0.0004	0.015
Regulation of anatomical structure size	472	22	10.0	0.0005	0.015

*N* numbers, *de novo autism* genes implicated in de novo autism studies, *de novo autism novel* newly curated set of genes implicated in de novo autism studies, *de novo* *schizophrenia novel* newly curated set of genes implicated in de novo schizophrenia studies, *P value adj* adjusted *p* value after Benjamini & Hochberg correction for multiple testing.

In the gene set enrichment analyses for the 139 genes (harboring rare non-synonymous variants predicted to be potentially/probably damaging by all applied prediction tools), none of the tested pathways showed a significant enrichment after correction for multiple testing. However, 10 gene sets showed a nominally significant enrichment. These included the de novo autism gene set from Goes et al.^22^, and the two larger, newly curated de novo autism and schizophrenia gene sets (Supplementary Table 3).

## Discussion

In the present WES investigation of 81 affected individuals from 27 multigenerational and multiply affected BD families, a total of 378 rare, non-synonymous, and potentially functional variants were carried by all three investigated members of at least one family. These variants were located in 368 genes. Eight of these genes harbored rare segregating variants in two independent families. In the extended segregation analysis, five of these genes carried variants with full or nearly full penetrance. The lack of formal statistical evidence in the RareIBD analysis was probably attributable to the limited sizes of the pedigrees.

These five genes included the brain-expressed genes *RGS12* and *NCKAP5*, which were considered the most promising BD candidates on the basis of independent evidence. The brain-expressed gene *RGS12* is located on chromosome 4p16.3, and belongs to the GTPase activating protein (GAP) family. GAPs are regulators of heterotrimeric G-proteins, and facilitate the hydrolyzing of the alpha subunits from GTP. The RGS proteins thereby drive G-proteins into an inactive GDP form, which results in the downregulation of GPCR signaling^60,61^. In a GWAS of 24,025 patients and controls, the present authors identified common variation in *ADCY2* as a risk factor for BD^8^. Interestingly, ADCY2 is regulated by heterotrimeric G-proteins, which provides further evidence for the involvement of this pathway in BD development^62^. *RGS12* is also of interest in terms of its functional role in the coordination of Ras-dependent signals, which are necessary for the promotion and/or maintenance of neuronal differentiation^63^. In addition, WES studies identified rare de novo missense mutations in *RGS12* in two independent schizophrenia patients (p.P518L and p.R702L)^52,64^. In view of the reported genetic overlap between schizophrenia and BD^13^, this finding renders *RGS12* a highly promising candidate in terms of follow-up analyses.

To date, *NCKAP5* has been implicated in GWAS of BD, schizophrenia, hypersomnia, personality traits, and mood states^65–68^. This cumulative evidence for an association with neuropsychiatric disease and other phenotypes, combined with the present WES finding, suggests that *NCKAP5* may contribute to the development and maintenance of a broad spectrum of psychiatric disorders, including BD. However, the function of *NCKAP5* remains unknown, which precludes speculation concerning biological processes of relevance to BD.

In the gene *DCAF5*, the same variant (p.R923Q) was found in two independent families (Table 2). Furthermore, in family 0009, the variant showed a nominally significant association in the RareIBD analysis (*p*_nom_ = 0.0097, *p*_corr_ = 0.1552). The *DCAF5* gene encodes the DDB1 and CUL4 associated factor 5. Rare chromosomal microdeletions involving *DCAF5* have been described in patients with intellectual disability, congenital heart defects, and facial dysmorphism^69^. However, no association with psychiatric disorders has yet been reported. A plausible hypothesis is that the identified variant has a higher allele frequency in the Spanish population than in the ExAC data used for the estimation of variant frequency in the present study. However, the kinship analysis confirmed the absence of any close genetic relationship between the 27 investigated families.

A comparison of the 368 genes implicated in the present study with those identified in previous BD GWAS and sequencing studies revealed a total of 19 overlapping genes (Table 3). Interestingly, these included several genes of relevance to the cytoskeleton, e.g., *ANK3*, *MACF1*, *MYO10*, and *SYNE1*^70^. This is consistent with the findings of previous studies, which suggested a potential contribution of cytoskeleton pathways to BD etiology^71,72^.

The 378 identified rare genetic variants showed significant enrichment in the de novo autism gene set retrieved from Goes et al.^22^ (*p*_adj_ < 0.006), as well as in the larger, newly curated de novo autism gene set (*p*_adj_ < 0.006). These results support the findings of the exome-sequencing study by Goes et al.^22^, which investigated 36 cases from eight multiply affected BD families. The present data and those of Goes et al. add to accumulating evidence of an etiological genetic overlap between BD and autism^73–75^.

For the three remaining gene sets retrieved from Goes et al.^22^, no significant enrichment was detected after correction for multiple testing. However, the analysis of the larger, newly curated de novo schizophrenia gene set revealed significant enrichment (*p*_adj_ = 0.015). This provides further evidence for a substantial genetic correlation between BD and schizophrenia at the rare-sequence-variant level^22^.

Of the 55 additional gene sets that have shown association with BD in previous studies, only “Regulation of anatomical structure size” showed a significant enrichment in the present study (*p*_adj_ = 0.015; Supplementary Table 3). This gene set was reported to be associated with BD in the pathway analysis of GWAS data performed by the Psychiatric Genomics Consortium^53^. The gene set contains 472 genes with an involvement in processes that modulate the size of anatomical structures^54,55^.

The aim of the present enrichment analysis was to enable a comprehensive investigation of gene sets with a previously reported association with BD in our 27 multiply affected families. The findings of previous studies are at least partially heterogeneous, as: (i) the previously reported associations are based on different datasets (i.e., common variants in GWAS vs. rare variants in sequencing studies); (ii) the various studies investigated individuals of different ethnicities; and (iii) the various sequencing studies applied different strategies, filter criteria, and enrichment analysis methods. This may be one reason for the relatively small overlap with previously reported BD associations found in the present study. Alternatively, this small overlap may be attributable to the presence of very pronounced inter-familial heterogeneity with respect to individual disease genes/pathways. To assess this, future analyses, involving uniform methods and filter strategies and large BD samples, are required.

The present WES step was restricted to BD cases only. The main reason for this approach was to facilitate data analysis, which had to take into account the different family structures. The possible presence of a candidate variant in unaffected family members was not an exclusion criterion, as available data on the genetic architecture of psychiatric disorders suggest that such variants have reduced penetrance^76^. To determine their degree of penetrance, a post hoc segregation analysis of the identified candidate variants was performed. The presence of a high-penetrance variant in a large proportion of older, unaffected individuals of a pedigree is unlikely. Of the 16 identified variants (Table 2), three were detected in several unaffected family members, which suggests that they are less likely to be highly penetrant. However, given the small number of affected and unaffected individuals on which they are based, estimations of penetrance must be viewed with great caution.

The power of the present study to detect strong association between rare variants and BD was limited. Previous authors have discussed potential determinants of the statistical power for calculating associations between a phenotype and rare variants, including study design, selected MAF cutoffs, and sample size^77,78^. To confirm the associations identified in the present study, replication studies and comprehensive analyses in additional, densely affected families are warranted. This could be achieved via combined analyses of exome-sequencing data in international Consortia, such as the Bipolar Sequencing Consortium^79^. To reduce genetic heterogeneity, investigation of families with an accumulation of distinct subphenotypes could also be considered. The investigation of such families might also generate novel insights into differences in the genetic background of diverse clinical presentations^18,80^.

The present analyses focused on rare variants (MAF < 0.1%) with strong predicted effects on protein function. As rare variants with moderate effects may also contribute to BD susceptibility, a second filtering step was performed using relaxed criteria with regard to the applied prediction tools (broader variant list in Supplementary Table 2). Future studies of a larger number of multiplex families are required to: (i) assess their relevance in terms of BD etiology; and (ii) investigate the potential contribution of rare and low-frequency variants with a higher MAF (0.1–5%) to the high prevalence of BD in these families.

A limitation of the present study was that the estimation of the MAFs was based on reference data from the ExAC^30^. These data include diverse ethnicities and represent a mixture of different populations with a focus on European samples, which should largely correspond to the ethnicity of the Spanish and German populations^81^. To determine the exact ethnicity of the investigated families, further analyses are necessary, e.g., a principal component analysis using genome-wide genotype data. The present authors are currently generating these data within the frame-work of a follow-up study.

In conclusion, rare and potentially functional variants identified in 27 multiply affected Spanish and German families implicated a total of 368 genes in BD etiology. Eight of these genes were detected in at least two independent families. The most promising variants were identified in the gene *RGS12*, which has been reported in previous next-generation sequencing studies of schizophrenia. Gene set analysis provided further evidence for a significant enrichment of rare segregating variants in genes reported in de novo studies of autism and schizophrenia, which suggests a possible genetic overlap of BD with autism and schizophrenia at the level of rare-sequence variants. The present data suggest novel BD candidate genes, and may contribute to an improved understanding of the biological basis of this common and often devastating disease.

## Supplementary information

Supplementary Table legends

Supplementary Figure legends

Supplementary Figure 1

Supplementary Figure 2

Supplementary Table 1

Supplementary Table 2

Supplementary Table 3
