# Supplementary material for: Whole-exome sequencing of 81 individuals from 27 multiply affected bipolar disorder families
Source: Transl Psychiatry. 2020 Feb 4;10:57. doi: 10.1038/s41398-020-0732-y (PMC7026119; doi:10.1038/s41398-020-0732-y)
Supplement: Supplementary file 1 — Supplementary Table legends [file 41398_2020_732_MOESM1_ESM.docx]

**Supplementary Table legends**

**Supplementary Table 1** List of the 378 rare (minor allele frequency <0.1%) variants that were shared by all three investigated individuals in a given family and predicted to be potentially/probably damaging by at least three of the five applied prediction tools. The analysis included insertion/deletions that were predicted to be damaging by at least one of three prediction tools, and nonsense variants that were classified as (probably) disease causing by the MutationTaster tool.

**Supplementary Table 2** Overview of rare variants (minor allele frequency <0.1%) that were predicted to be potentially/probably damaging by at least one of the applied prediction tools. Variants listed in Supplementary Table 2 were shared by all three exome sequenced individuals in a given family.

**Supplementary Table 3** Overview of the gene set enrichment results for all 61 pathways tested in the present study.
