# Supplementary material for: Whole-exome sequencing of 81 individuals from 27 multiply affected bipolar disorder families
Source: Transl Psychiatry. 2020 Feb 4;10:57. doi: 10.1038/s41398-020-0732-y (PMC7026119; doi:10.1038/s41398-020-0732-y)
Supplement: Supplementary file 2 — Supplementary Figure legends [file 41398_2020_732_MOESM2_ESM.docx]

**Supplementary Figure legends**

**Supplementary Figure 1** Pedigrees of the 27 investigated Spanish and German families. a) Pedigrees investigated in the whole exome sequencing (WES) and extended segregation analysis; b) Pedigrees involved in WES only. WES was performed on subjects indicated by an arrow. Yellow indicates bipolar disorder (BD). Blue indicates other psychiatric phenotypes (e.g., recurrent major depressive disorder (MDD), single depressive episode or substance abuse). Unaffected individuals are indicated by white diamonds. In the families that were investigated in the extended segregation analysis step, the availability of DNA for individuals is depicted by a diamond with a central dot. To preserve the anonymity and confidentiality of the families, no information concerning sex or mortality is shown.

**Supplementary Figure 2** Detailed analytical plan of the present study. Abbreviations: BD, bipolar disorder; ExAC, Exome Aggregation Consortium; GTEx, Genotype-Tissue Expression database; GWAS, genome-wide association study; MAF, minor allele frequency; VCF, Variant Calling Files; WES, whole exome sequencing; WGS, whole genome sequencing.
