# Supplementary material for: Whole-exome sequencing of 81 individuals from 27 multiply affected bipolar disorder families
Source: Transl Psychiatry. 2020 Feb 4;10:57. doi: 10.1038/s41398-020-0732-y (PMC7026119; doi:10.1038/s41398-020-0732-y)

a) Pedigrees investigated in WES and extended segregation analysis

WES

Bipolar disorder

Unaffected

Other psychiatric phenotype (recurrent major depressive disorder, single depressive episode, depression not otherwise specified, substance abuse, suicide)

DNA available and included in segregation analysis

Family 0009

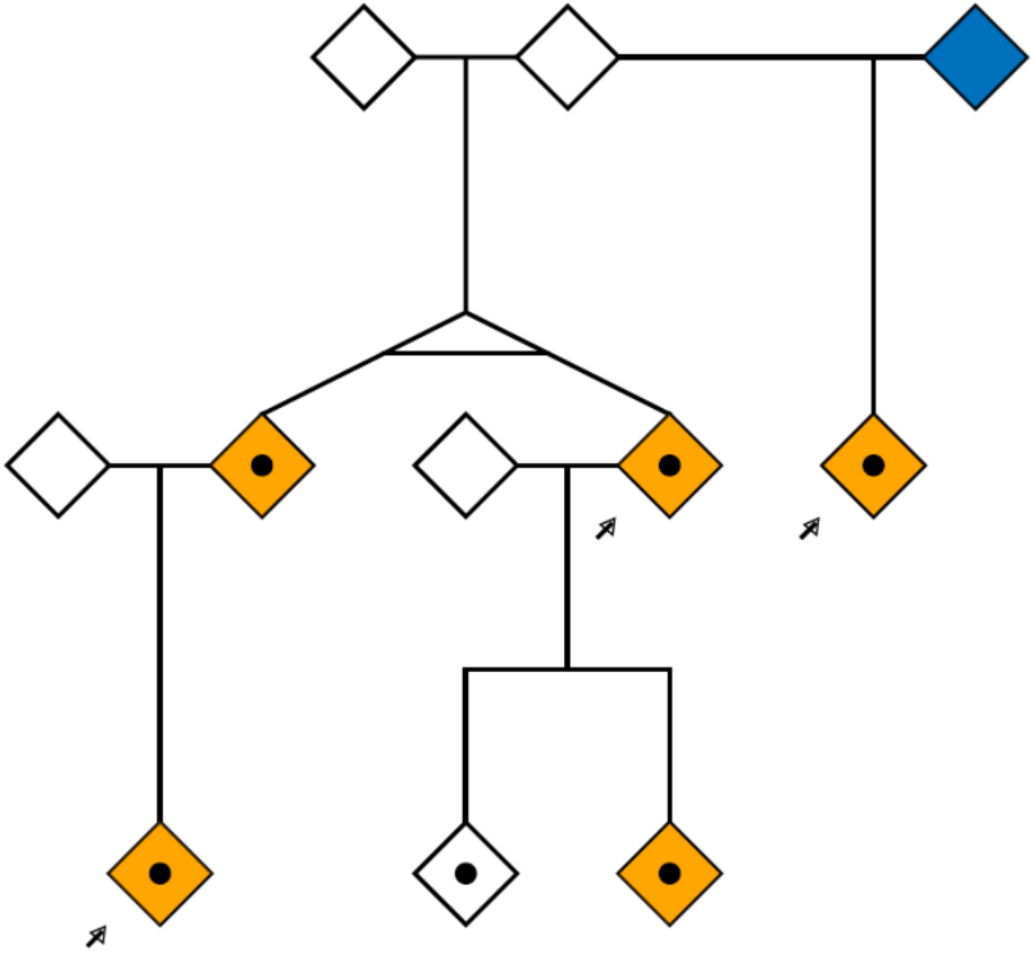

Family 0010

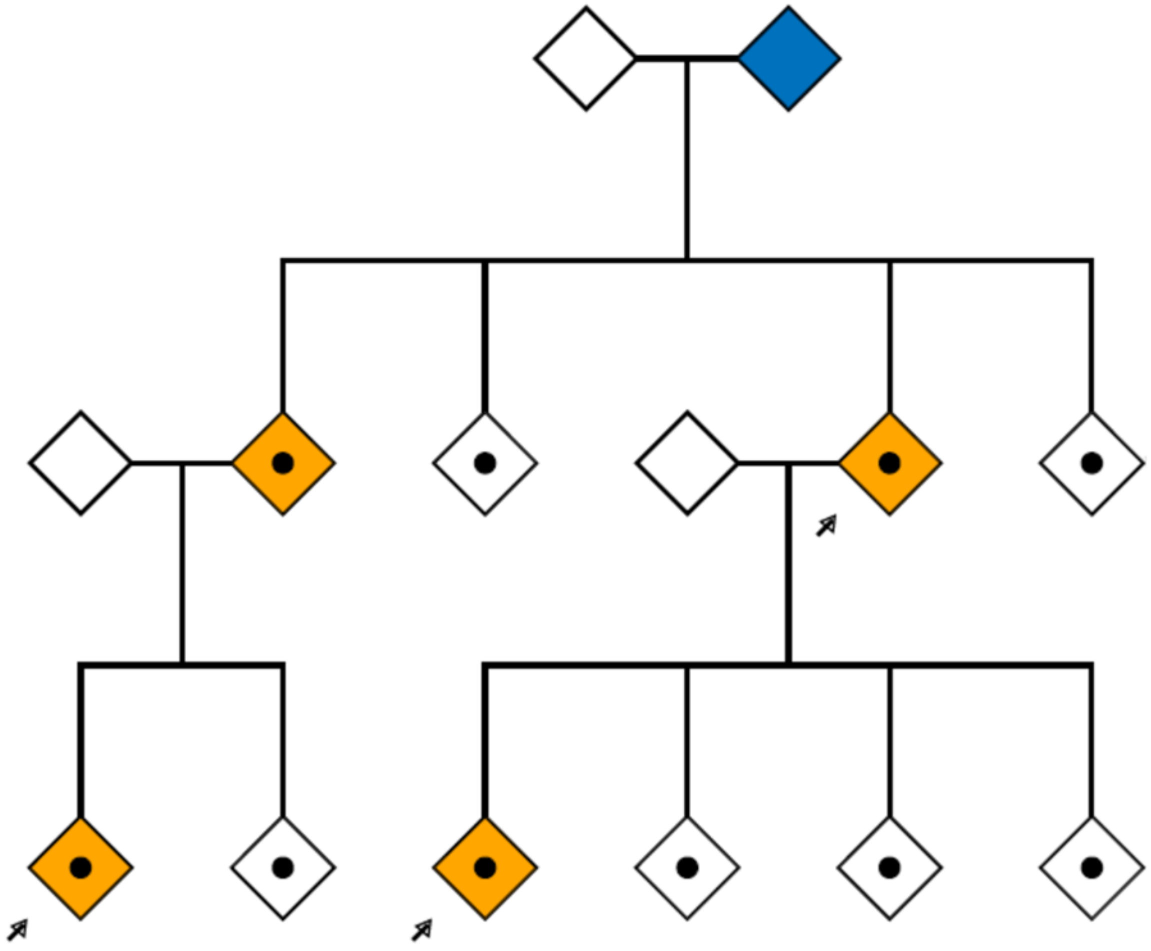

Family 0012

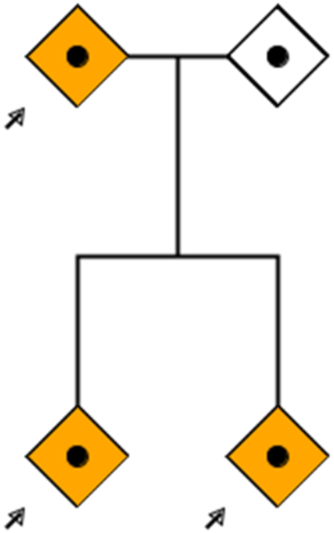

Family 0014

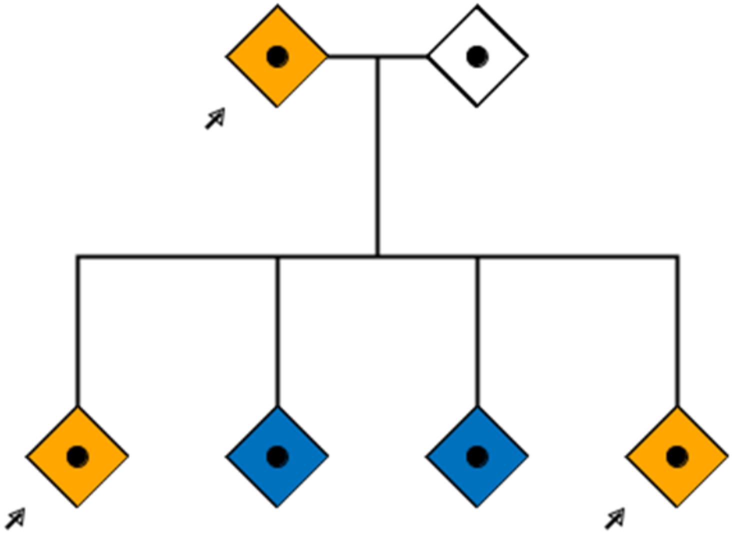

Family 0022

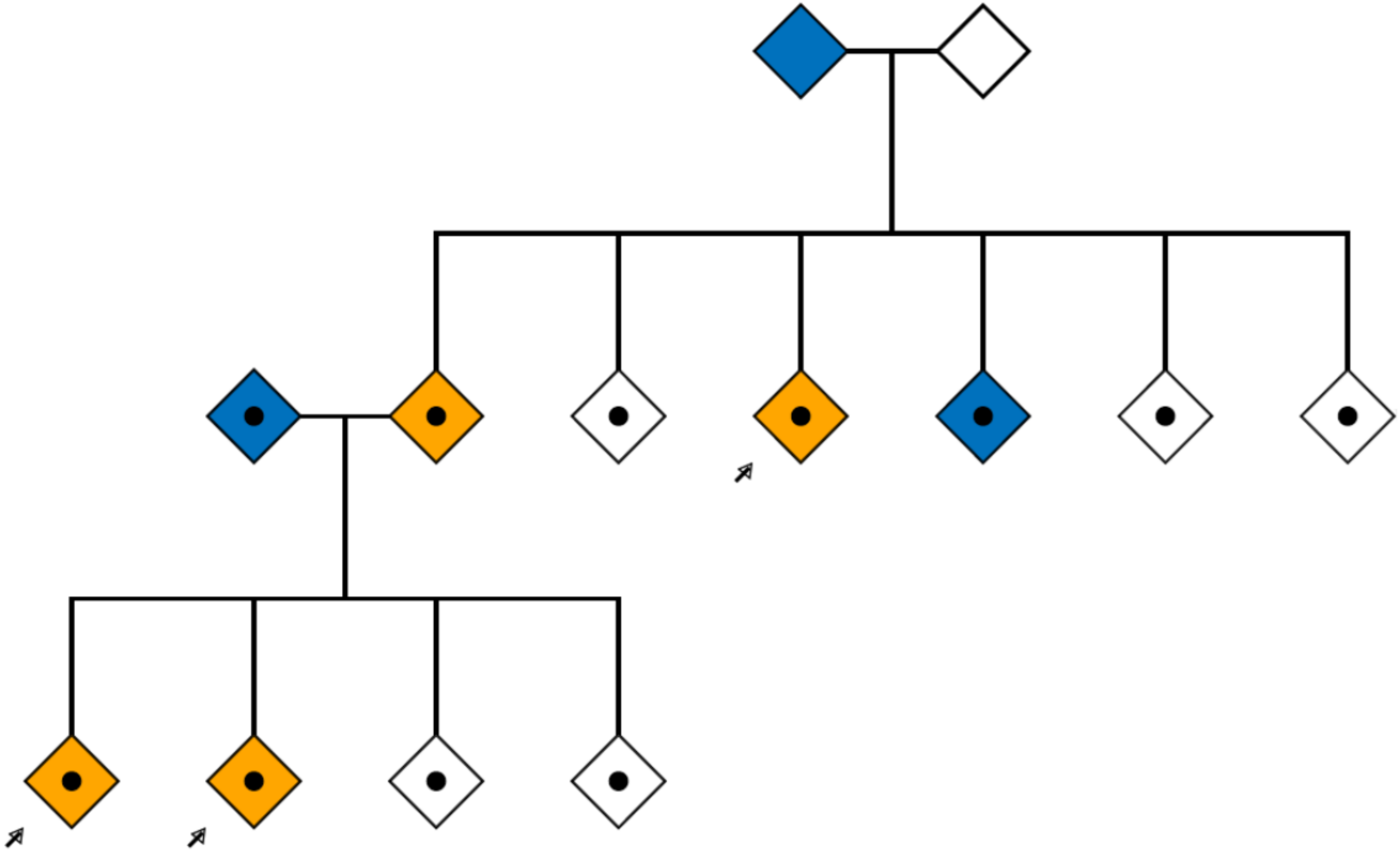

Family 0041

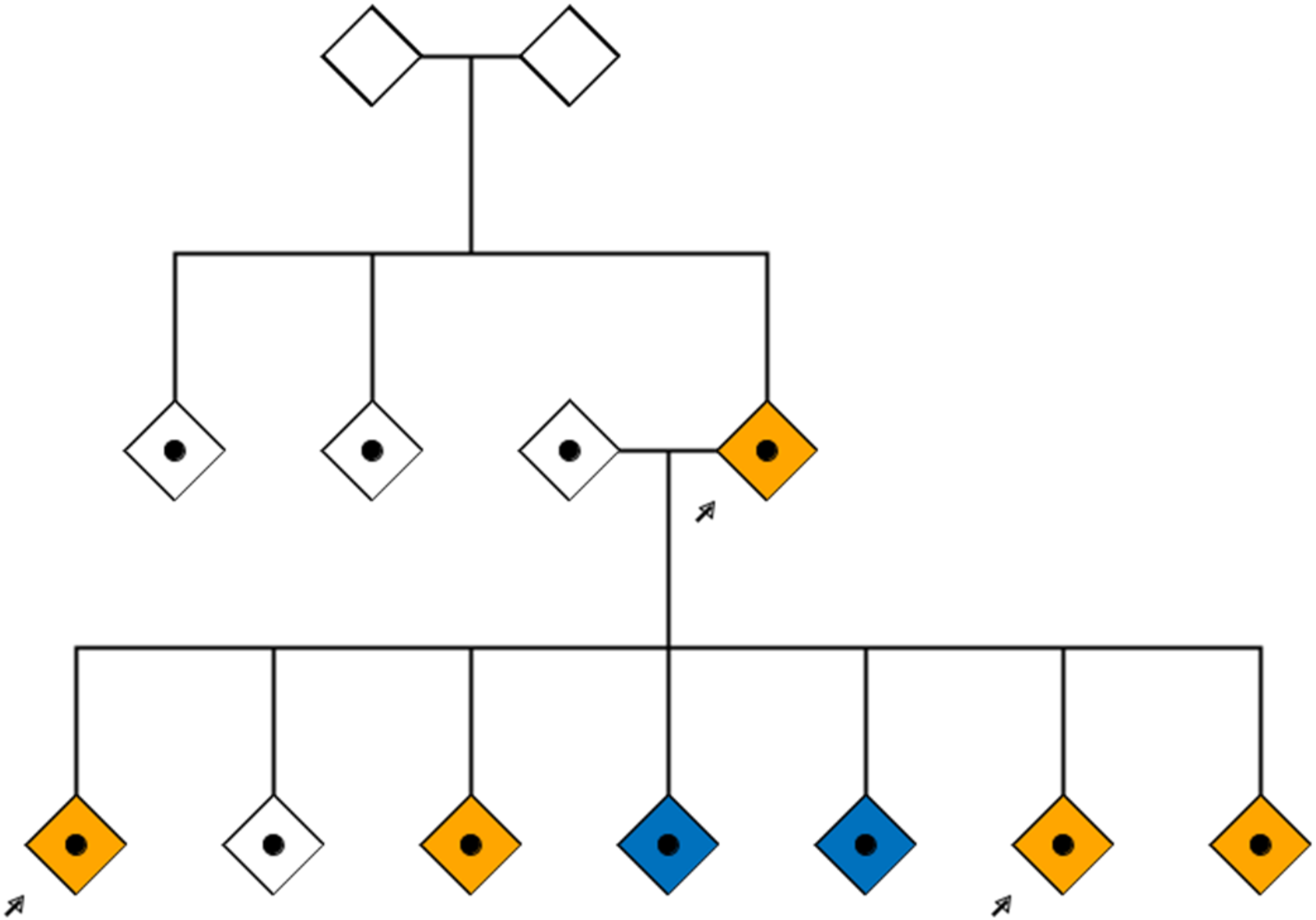

Family 0045

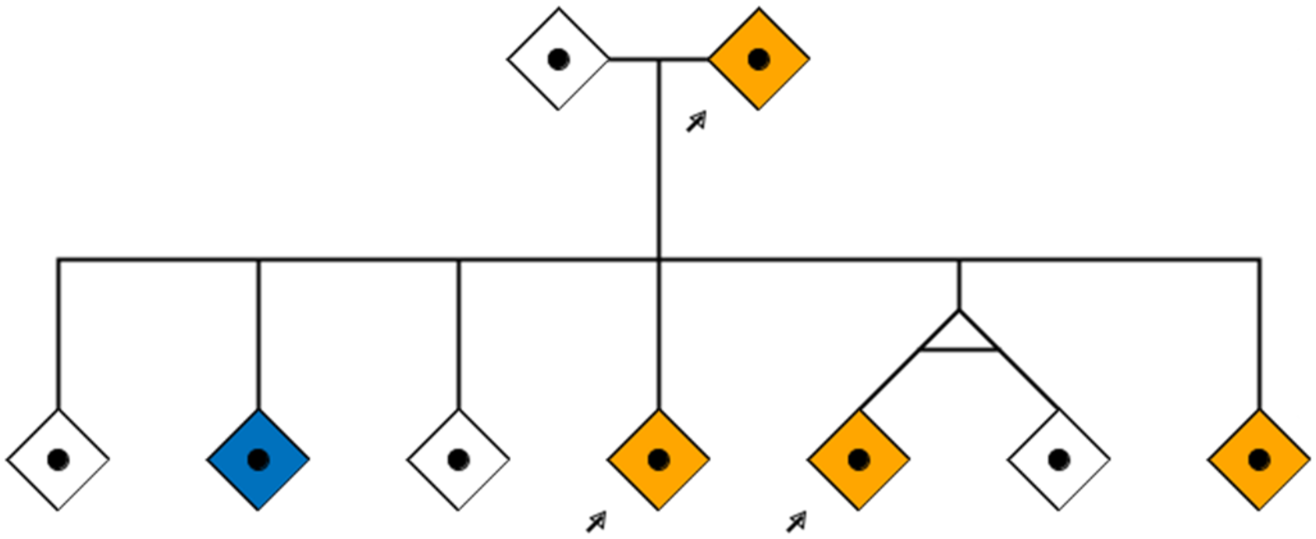

Family 0074

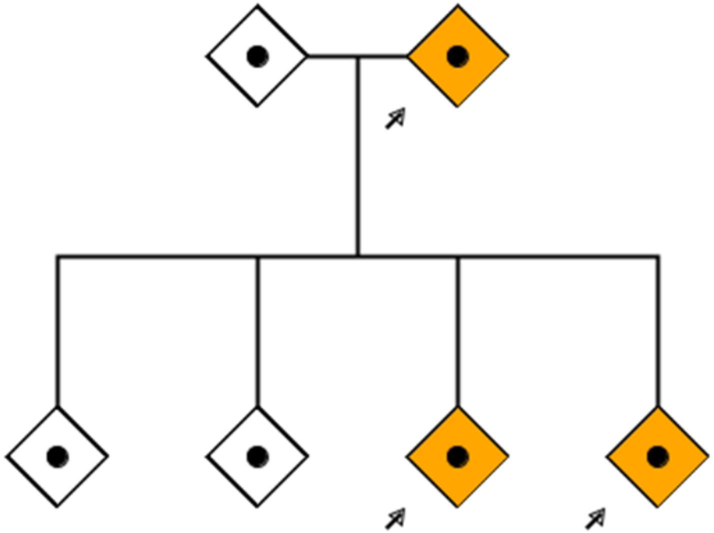

Family 0085

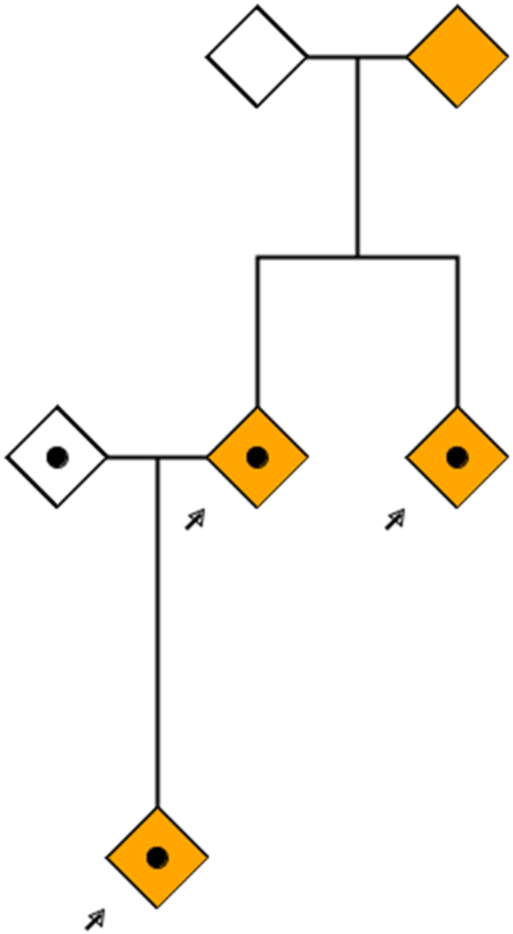

Family 0092

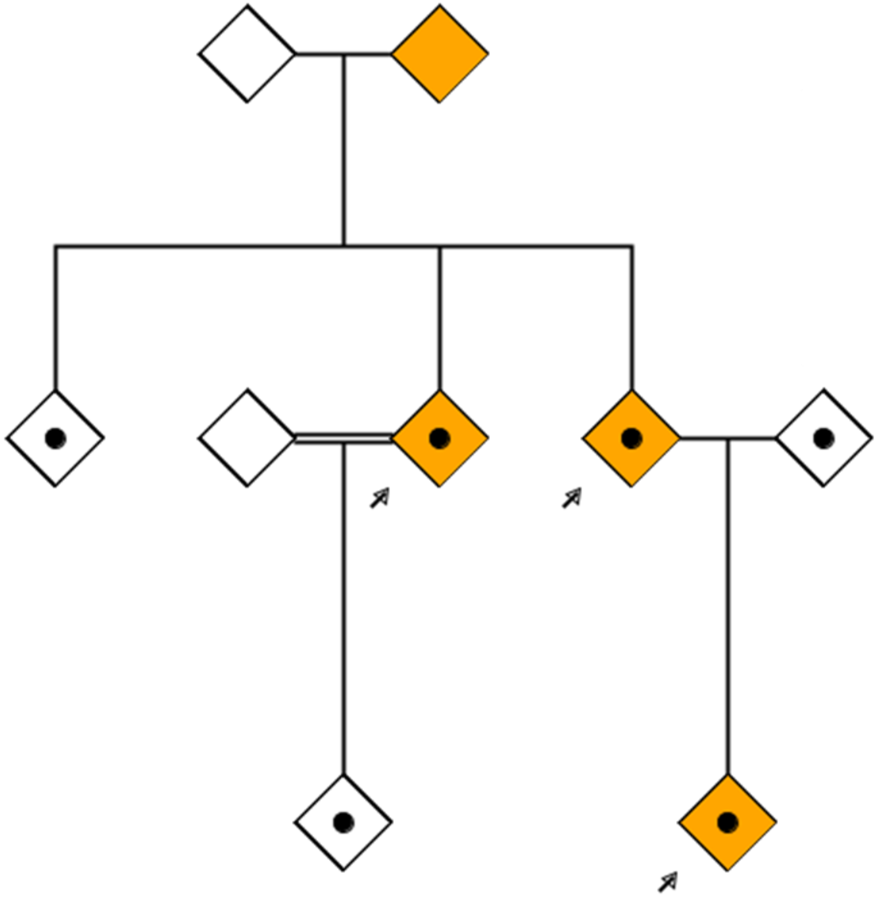

Family 0109

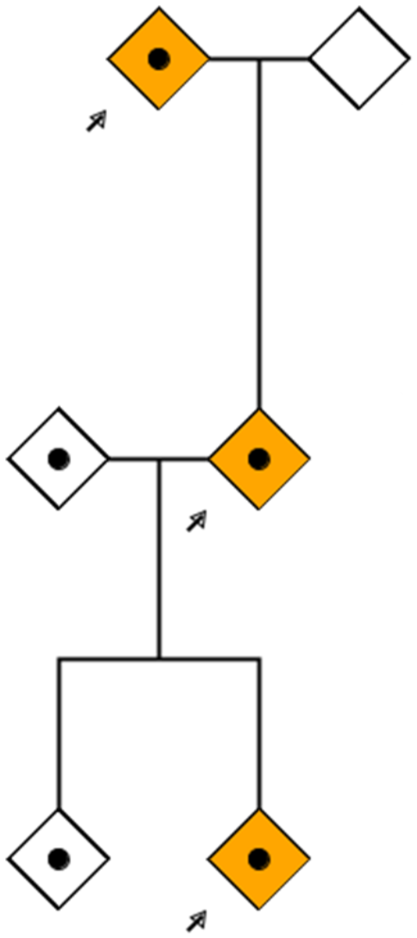

Family 1044

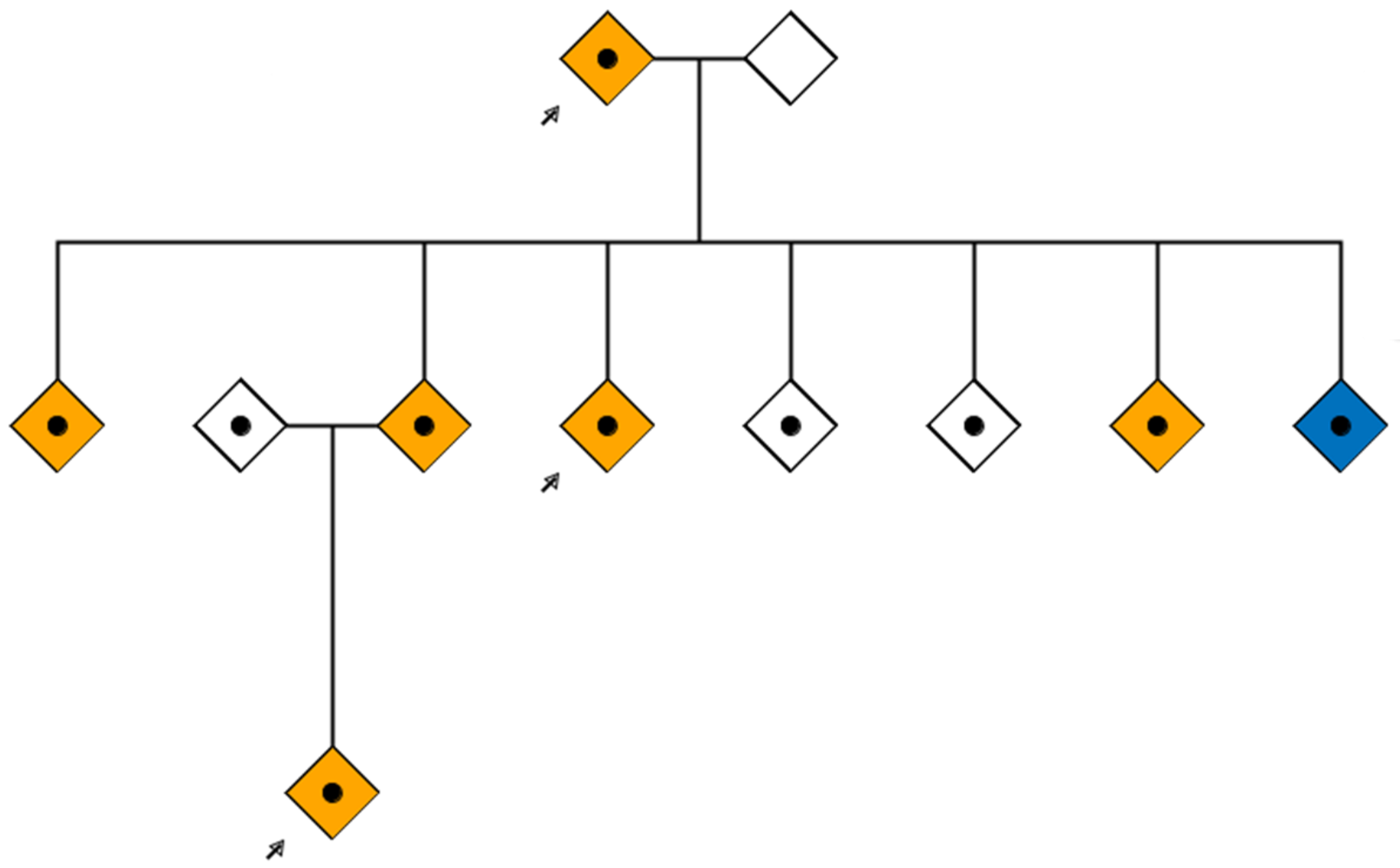

b) Pedigrees involved in WES only

WES

Bipolar disorder

Unaffected

Other psychiatric phenotype (recurrent major depressive disorder, single depressive episode, depression not otherwise specified, substance abuse)

Family 0001

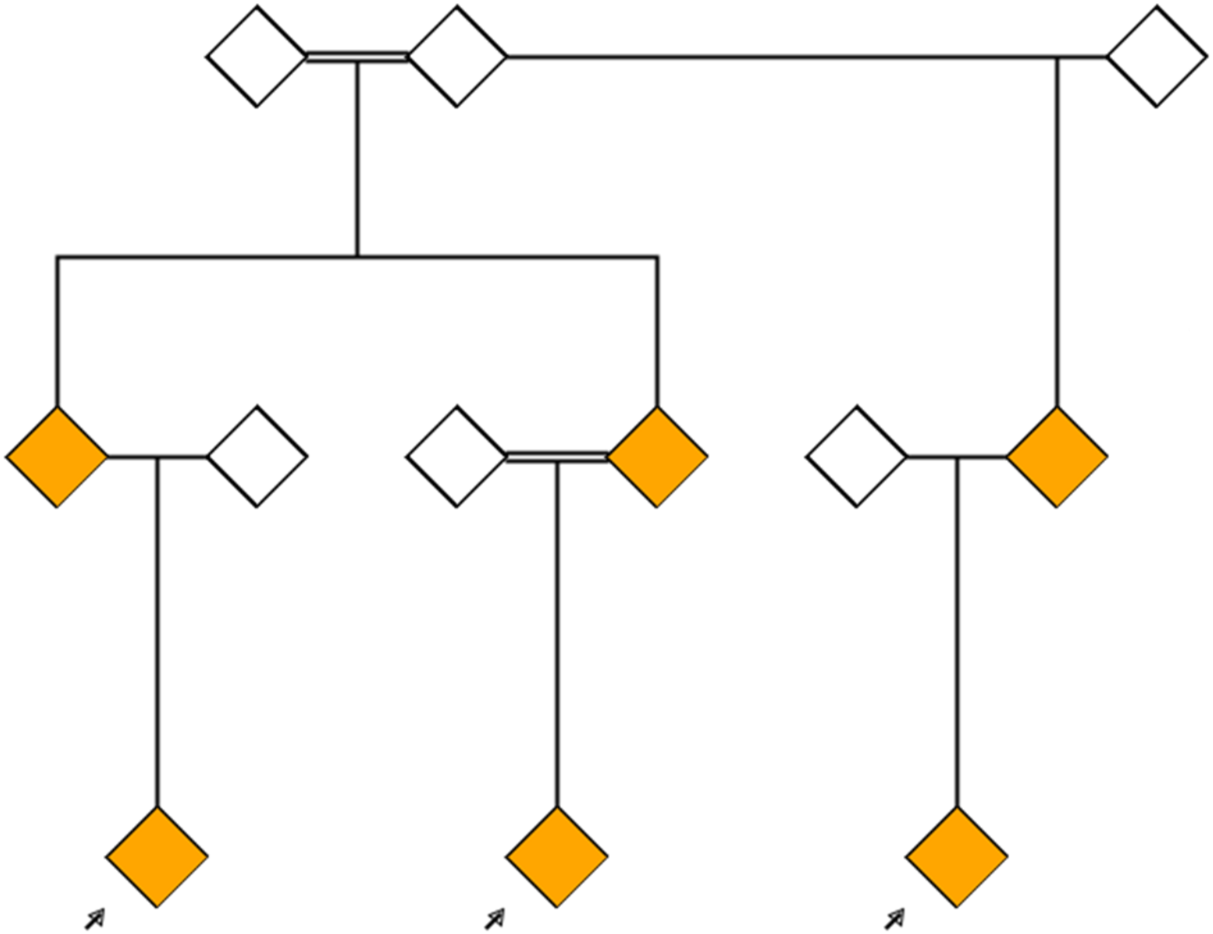

Family 0002

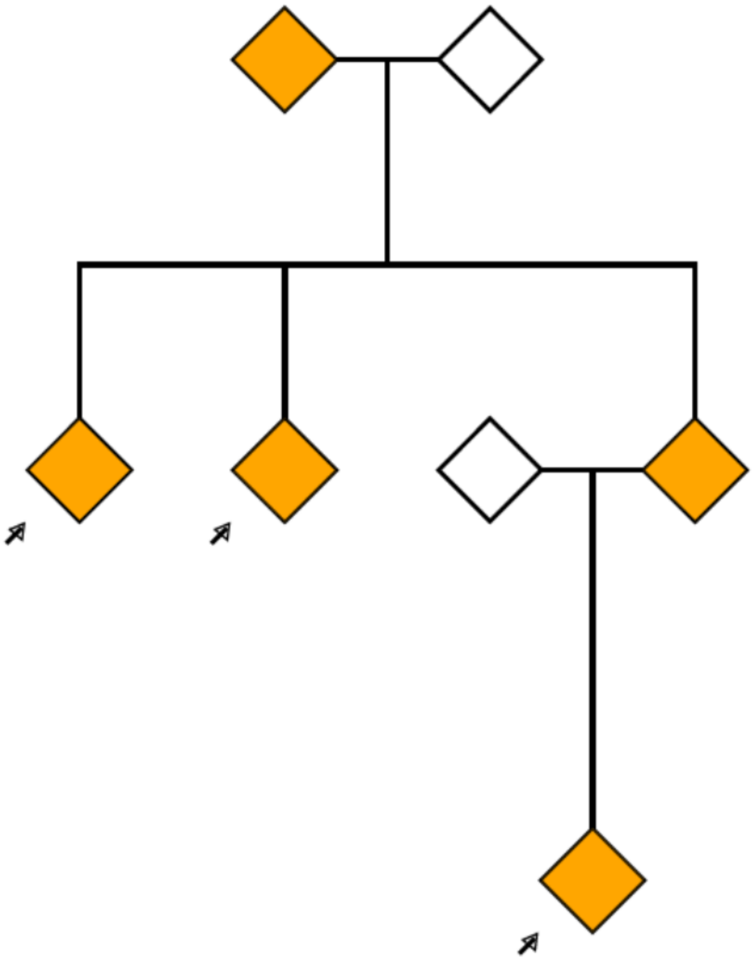

Family 0008

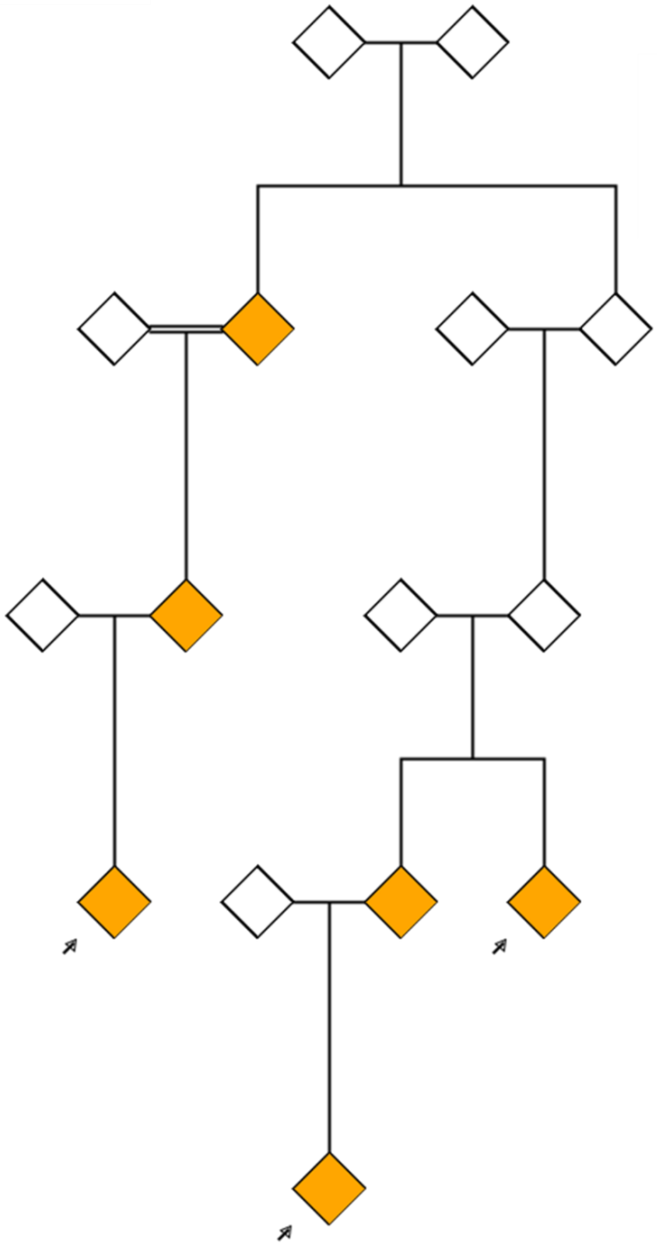

Family 0011

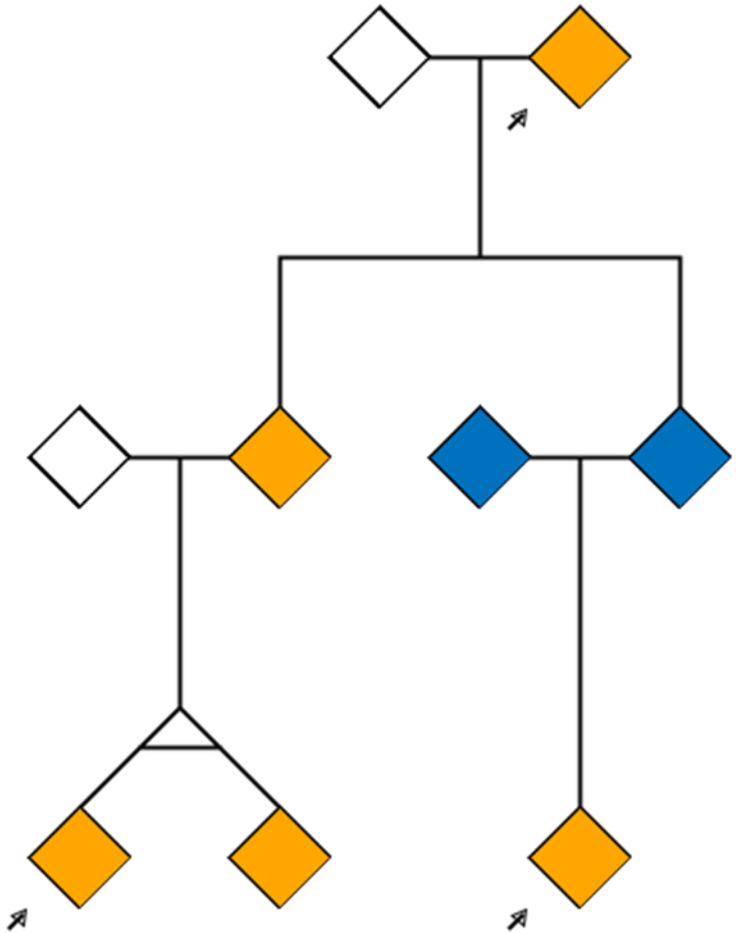

Family 0013

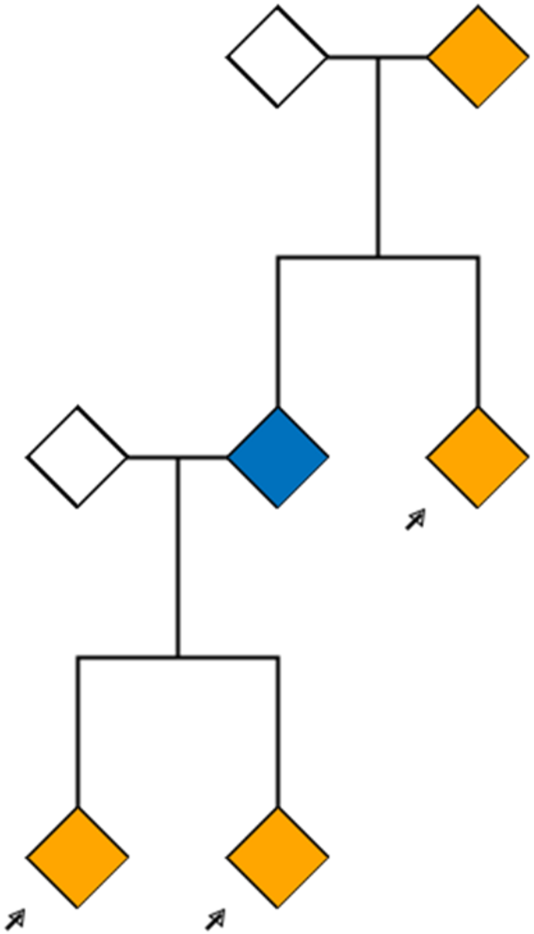

Family 0023

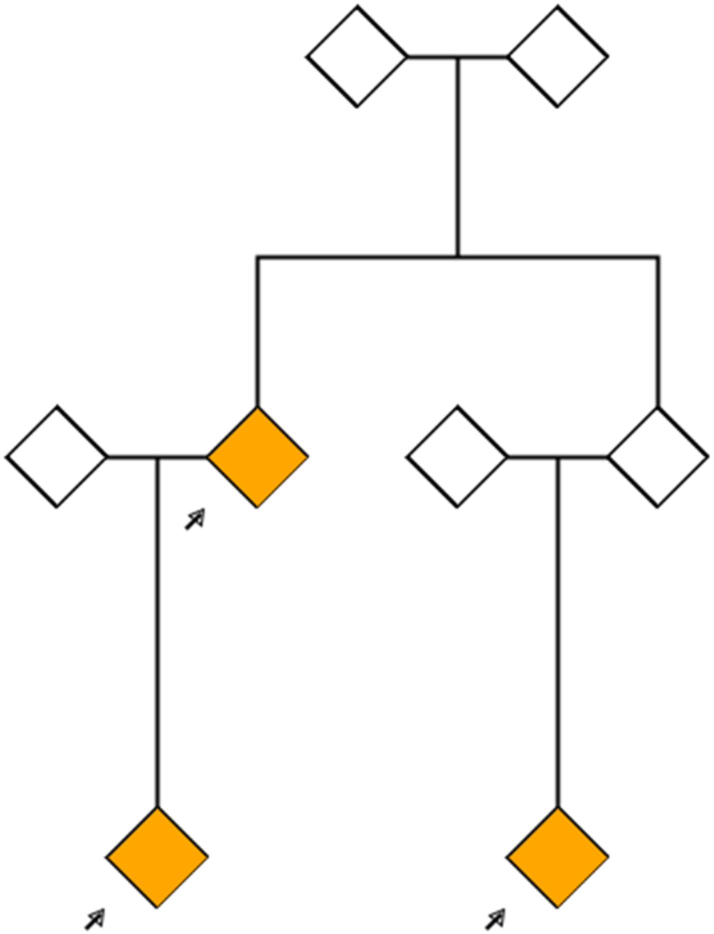

Family 0025

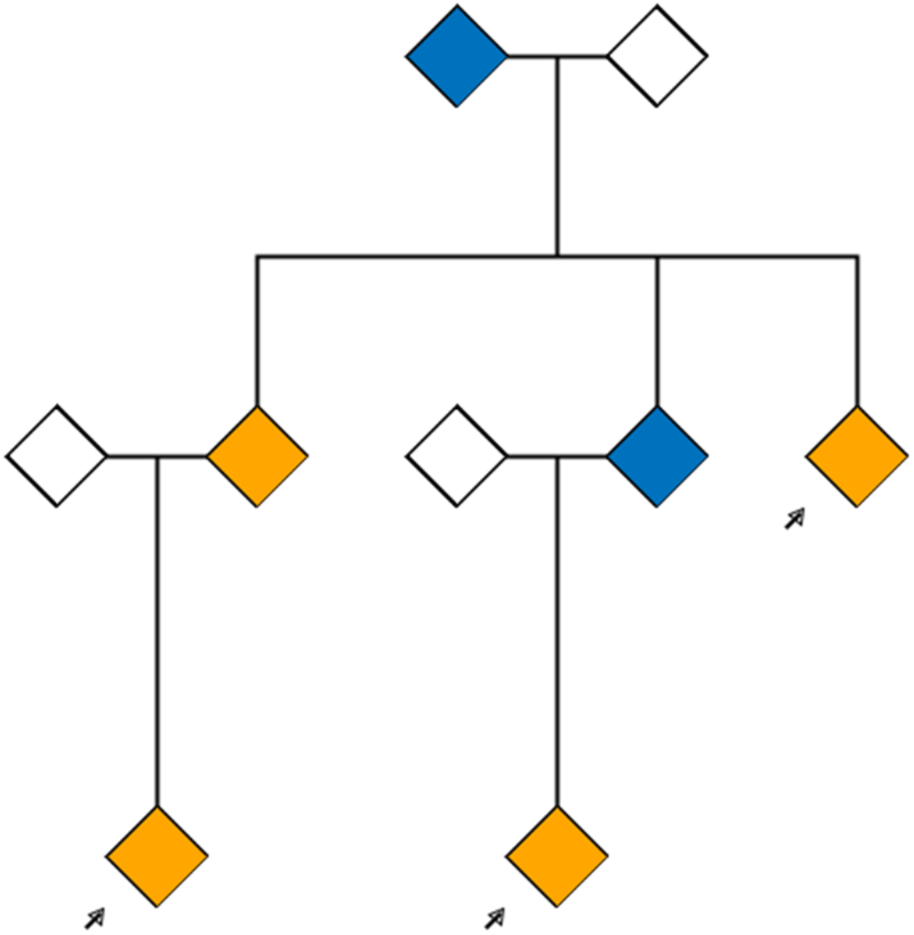

Family 0026

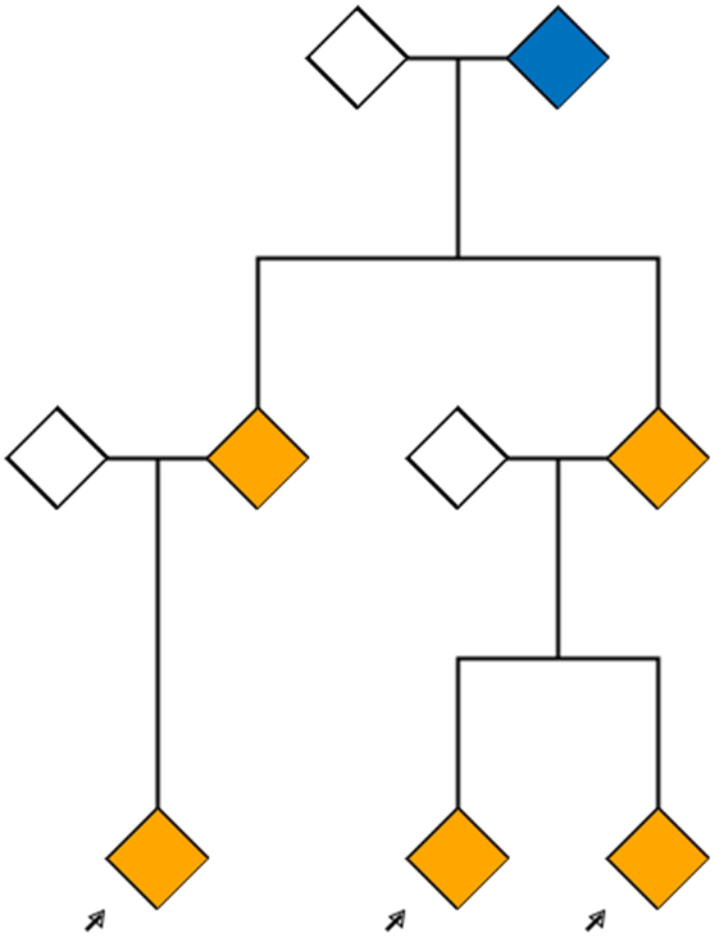

Family 0032

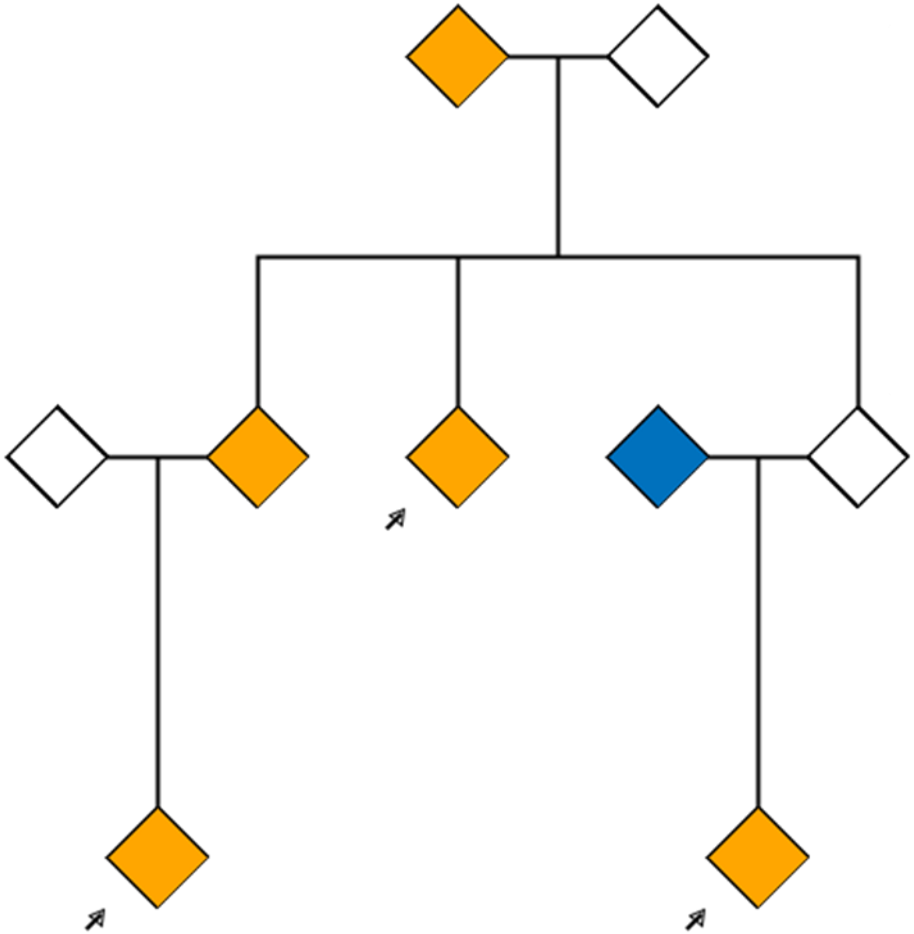

Family 0039

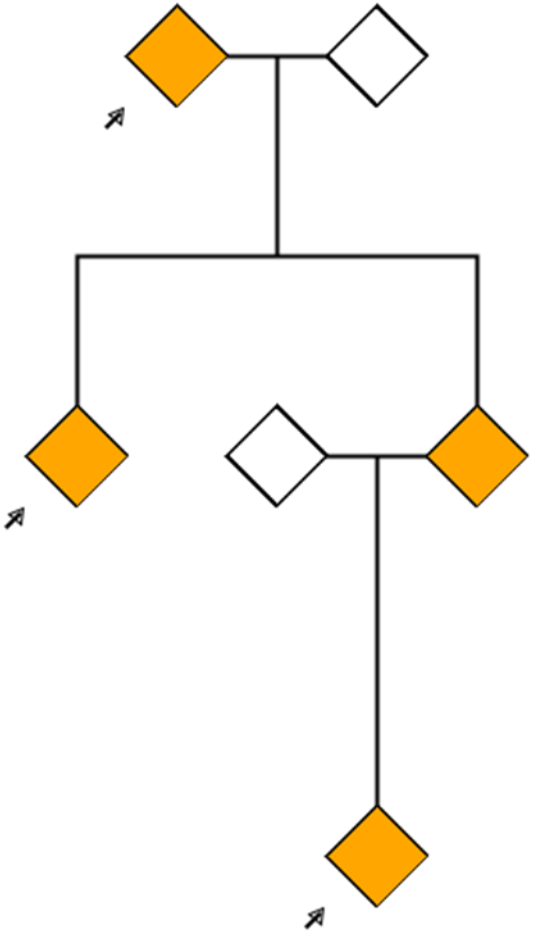

Family 0062

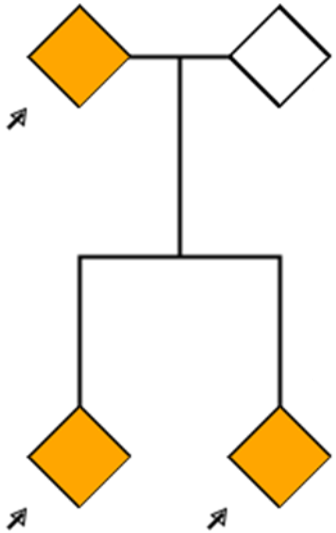

Family 0065

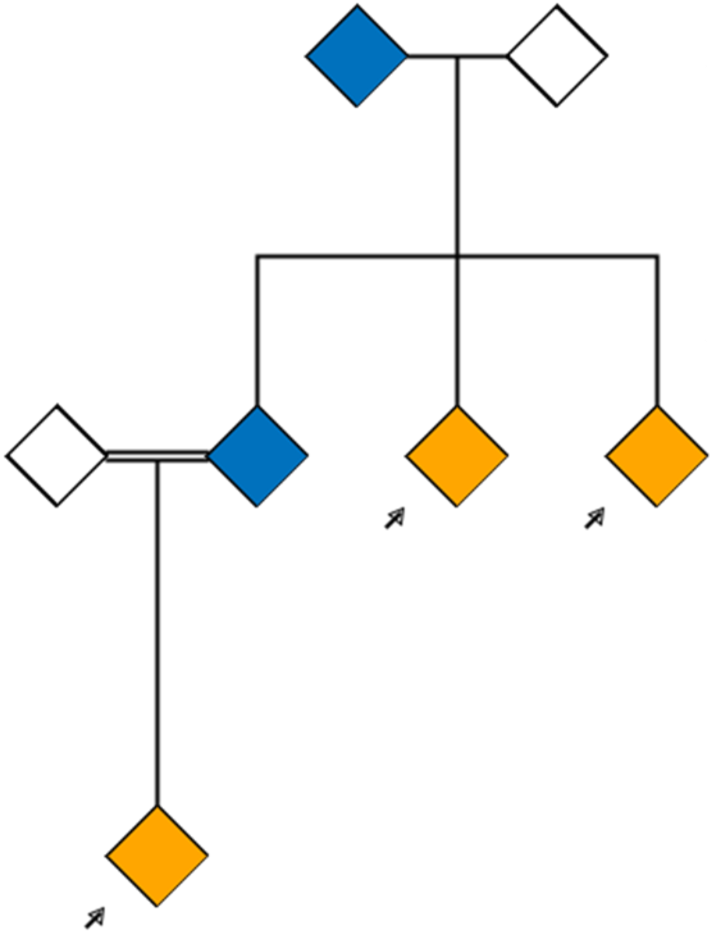

Family 0083

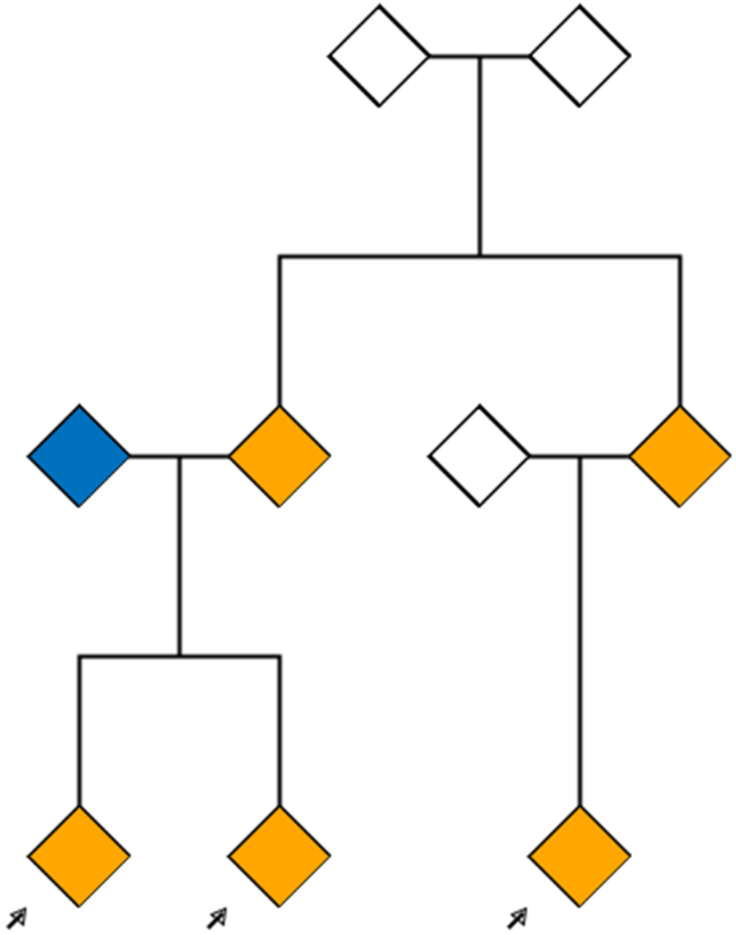

Family 0089

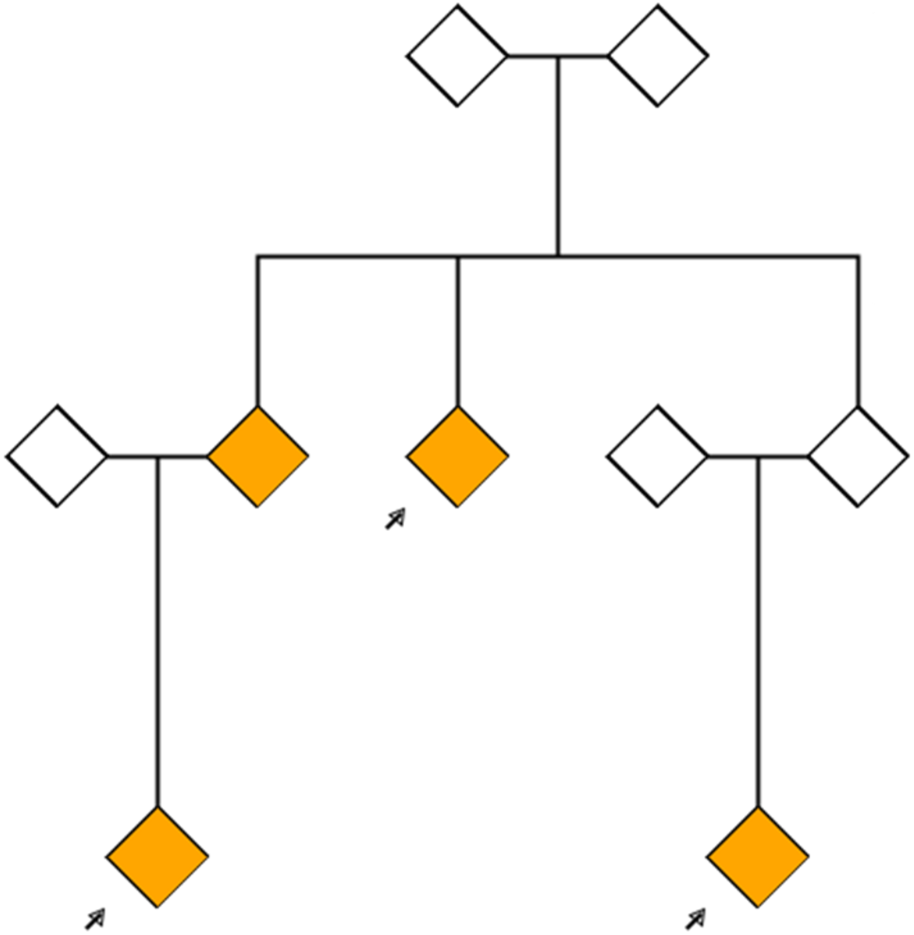

Family 0215

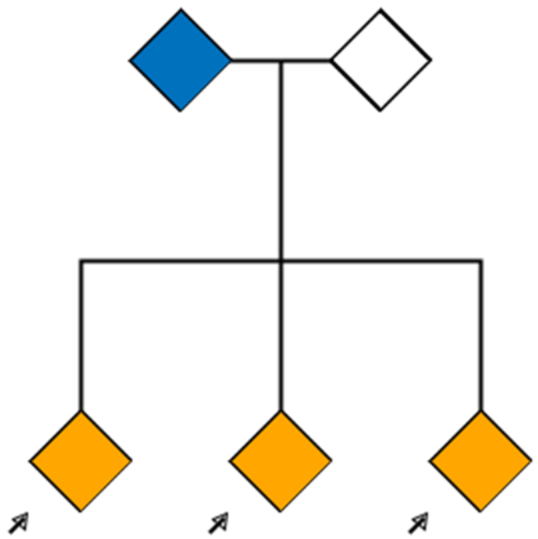

Supplement: Supplementary file 3 — Supplementary Figure 1 [file 41398_2020_732_MOESM3_ESM.pdf]
