# Supplementary material for: Whole-exome sequencing of 81 individuals from 27 multiply affected bipolar disorder families
Source: Transl Psychiatry. 2020 Feb 4;10:57. doi: 10.1038/s41398-020-0732-y (PMC7026119; doi:10.1038/s41398-020-0732-y)
Supplement: Supplementary file 4 — Supplementary Figure 2 [file 41398_2020_732_MOESM4_ESM.pdf]

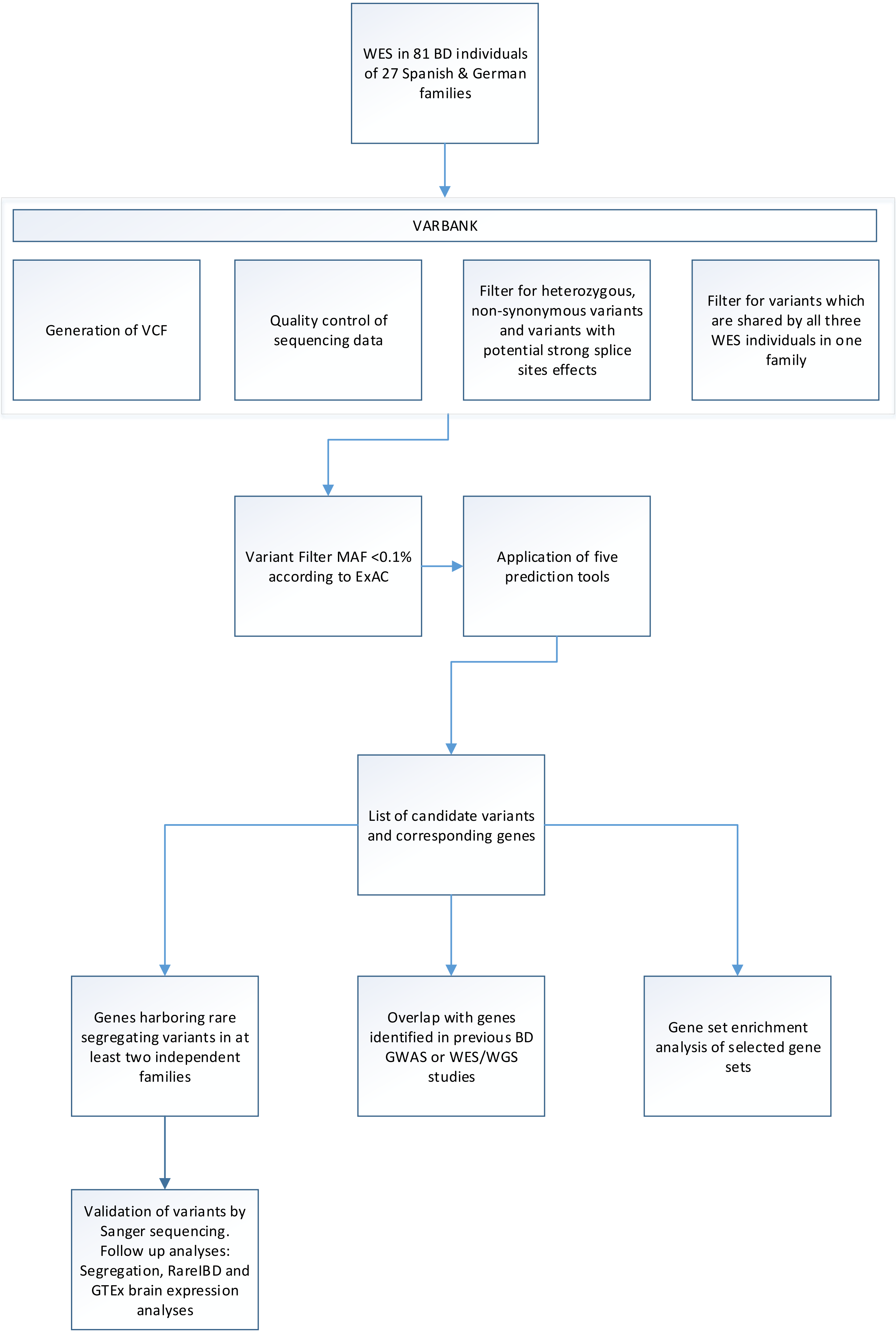
WES in 81 BD individuals of 27 Spanish & German families
VARBANK
Generation of VCF
Quality control of sequencing data
Filter for heterozygous, non-synonymous variants and variants with potential strong splice sites effects
Filter for variants which are shared by all three WES individuals in one family
Variant Filter MAF <0.1% according to ExAC
Application of five prediction tools
List of candidate variants and corresponding genes
Genes harboring rare segregating variants in at least two independent families
Overlap with genes identified in previous BD GWAS or WES/WGS studies
Gene set enrichment analysis of selected gene sets
Validation of variants by Sanger sequencing. Follow up analyses: Segregation, RareIBD and GTEx brain expression analyses
